# Crystal structure of *N*-[2-(cyclo­hexyl­sulfan­yl)eth­yl]quinolinic acid imide

**DOI:** 10.1107/S2056989017012142

**Published:** 2017-08-25

**Authors:** Hyunjin Park, Myong Yong Choi, Cheol Joo Moon, Tae Ho Kim

**Affiliations:** a Department of Chemistry (BK21 plus) and Research Institute of Natural Sciences, Gyeongsang National University, Jinju 52828, Republic of Korea

**Keywords:** crystal structure, theoretical calculations, quinolinic acid imide, hydrogen bonding

## Abstract

The title compound, C_15_H_18_N_2_O_2_S {systematic name: 6-[2-(cyclo­hexyl­sulfan­yl)eth­yl]-5*H*-pyrrolo­[3,4-*b*]pyridine-5,7(6*H*)-dione}, was obtained from the reaction of pyridine-2,3-di­carb­oxy­lic anhydride (synonym: quinolinic anhydride) with 2-(cyclo­hexyl­sulfan­yl)ethyl­amine. The dihedral angle between the mean plane of the cyclo­hexyl ring and the quinolinic acid imide ring is 25.43 (11)°. In the crystal, each mol­ecule forms two C—H⋯O hydrogen bonds and one weak C—O⋯π [O⋯ring centroid = 3.255 (2) Å] inter­action with neighbouring mol­ecules to generate a ladder structure along the *b*-axis direction. The ladders are linked by weak C—O⋯π [O⋯ring centroid = 3.330 (2) Å] inter­actions, resulting in sheets extending parallel to the *ab* plane. The mol­ecular structure is broadly consistent with theoretical calculations performed by density functional theory (DFT).

## Abstract 2

In the crystal of the title compound, C—H⋯O hydrogen bonds and C—O⋯π inter­actions form a two-dimensional network lying parallel to the *ab* plane.

## Chemical context   

Quinolinic anhydrides have been used extensively as versatile inter­mediates in the synthesis of various heterocyclic systems, such as aphthyridines, nicotinamides and isotonic derivatives. Recently, they have been exploited in anti­viral, dementia, anti-allergy and anti­tumor targets (Metobo *et al.*, 2013 ▸). In addition, it is expected that various metal complexes may be formed because they are composed of N/S-donor atoms. In particular, our group reported copper(I) coordination polymers with N/S-donor-atom ligands, which showed their various luminescence and reversible/irreversible structural transformations (Jeon *et al.*, 2014 ▸; Cho *et al.*, 2015 ▸). As part of our ongoing studies in this area, we designed and synthesized a new N/S-donor ligand, namely *N*-[2-(cyclo­hexyl­sulfan­yl)ethyl]quinolinic acid imide, which was prepared from the reaction of quinolinic anhydride with 2-(cyclo­hexyl­sulfan­yl)ethyl­amine. Herein, we report its crystal structure.
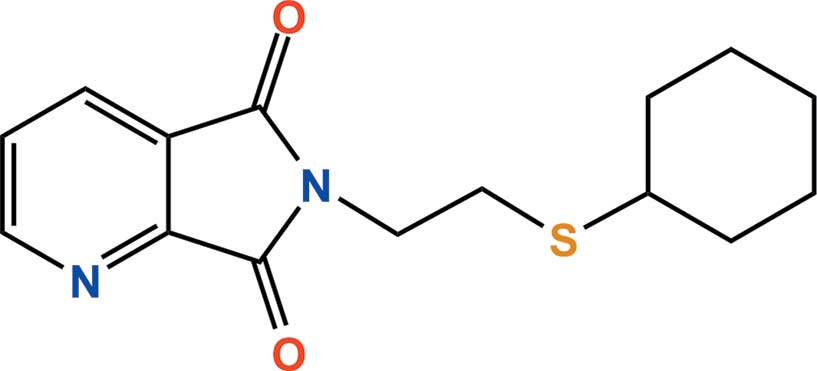


## Structural commentary   

**Figure 1:**
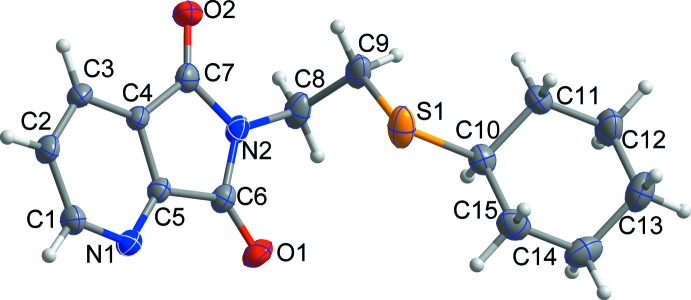
The mol­ecular structure of the title compound, with displacement ellipsoids drawn at the 50% probability level.

The crystal structure of the title compound is shown in Fig. 1 ▸. The cyclo­hexyl ring adopts a chair conformation, with the exocyclic C—S bond in an equatorial orientation; the dihedral angle between the mean plane (r.m.s. deviation = 0.2317 Å) of the cyclo­hexyl ring and the quinolinic acid imide ring is 25.43 (11)°. All bond lengths and angles are normal and comparable to those observed in similar crystal structures (Garduño-Beltrán *et al.*, 2009 ▸; Inoue *et al.*, 2009 ▸).

## Supra­molecular features   

**Figure 2:**
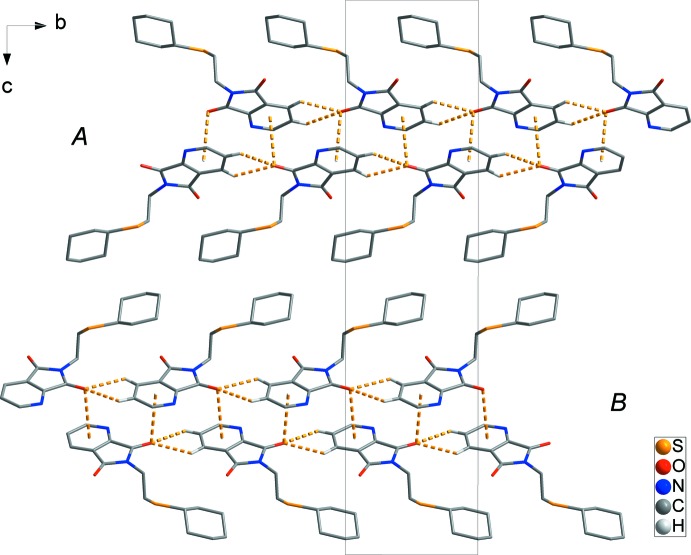
The crystal packing of the title compound, indicating the C—H⋯O hydrogen bonds and C—O⋯π inter­actions (yellow dashed lines) [symmetry codes: (i) *x*, *y* + 1, *z*; (ii) 1 − *x*, −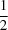 + *y*, 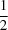 − *z*], which results in a one-dimensional ladder structure along the *b* axis.

**Figure 3:**
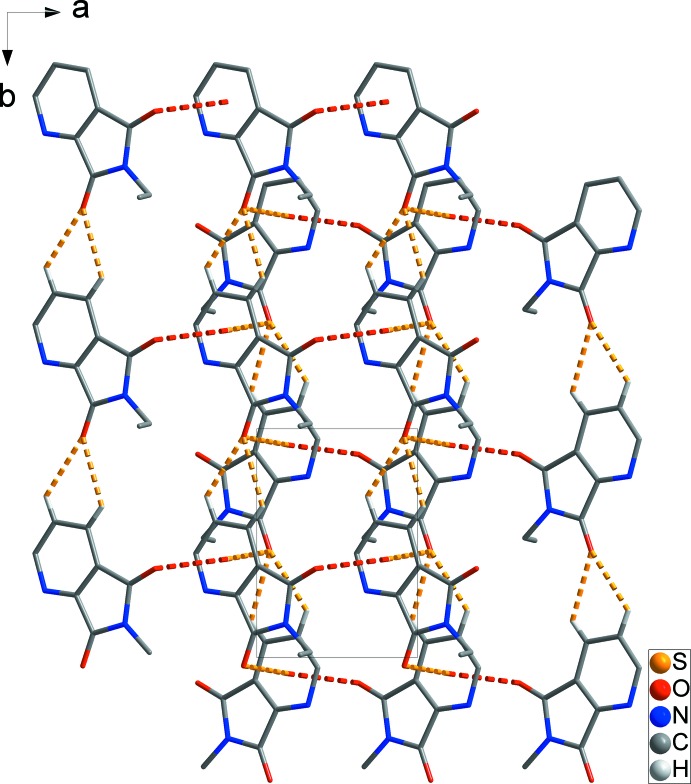
The packing diagram, showing the two-dimensional network structure formed by C—O⋯π inter­actions (red dashed lines) [symmetry code: (iii) −1 + *x*, *y*, *z*]. H atoms and cyclo­hexa­nesulfanyl groups not involved in inter­molecular inter­actions have been omitted for clarity.

In the crystal, mol­ecules are linked by C2—H2⋯O1^i^ and C3—H3⋯O1^i^ hydrogen bonds [H⋯O = 2.50 and 2.55 Å, respectively; symmetry code: (i) *x*, *y* + 1, *z*; Table 1 ▸], and weak C6—O1⋯*Cg*1^ii^ (*Cg*1 is the centroid of the N1/C1–C5 ring) inter­actions [O⋯π = 3.255 (2) Å; symmetry code: (ii) 1 − *x*, −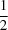 + *y*, 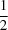 − *z*], forming a one-dimensional ladder structure along the *b* axis. The ladders are packed in an *ABAB* pattern along the *c* axis (yellow dashed lines in Fig. 2 ▸). In addition, the ladders are linked by C7—O2⋯*Cg*1^iii^ inter­actions [O⋯π = 3.330 (2) Å; symmetry code: (iii) −1 + *x*, *y*, *z*], resulting in the formation of a two-dimensional network structure lying parallel to the *ab* plane (red dashed lines in Fig. 3 ▸).

## Theoretical calculations   

To support the experimental data based on the diffraction study, computational calculations on the *N*-[2-(cyclo­hexyl­sulfan­yl)eth­yl]quinolinic acid imide mol­ecule were performed using the *GAUSSIAN09* software package (Frisch *et al.*, 2009 ▸). Full geometry optimizations were calculated at the DFT level of theory using a basis set of 6-311++G(d,p). The optimized parameters, such as bond lengths and angles, are in generally good agreement (the largest bond-length deviation is less than 0.03 Å) with the experimental crystallographic data (Table 2 ▸). The calculated and experimental torsion angles for N2—C8—C9—S1 (C8—C9—S1—C10) are 53.64 (65.80) and 64.2 (3)° [97.4 (2)°], respectively. The calculated and experimental dihedral angle between the ring systems were 25.34 and 25.43 (11)°, respectively. However, several relatively large differences between the experimental and theoretical data (see Table 2 ▸) may be due to the packing effects induced by the inter­molecular inter­actions in the crystal.

## Synthesis and crystallization   

A mixture of quinolinic anhydride (0.67 g, 5.0 mmol) and 2-(cyclo­hexyl­sulfan­yl)ethyl­amine (0.83 g, 5.3 mmol) in toluene (15 ml) was heated at 433 K with stirring for 8 h. The crude product was extracted with di­chloro­methane. The di­chloro­methane layer was dried with anhydrous Na_2_SO_4_ and evaporated to give a crude solid. The reaction mixture was then concentrated and purified by chromatography on silica gel (MeCOOEt/*n*-C_6_H_14_ = 30/70 *v*/*v*, *R*_F_ = 0.28) (Kang *et al.*, 2015 ▸). Colourless plates were obtained by slow evaporation of a hexane solution of the title compound. ^1^H NMR (300 MHz, CDCl_3_): δ 7.40 (*dd*, H, Py), 8.02 (*t*, H, Py), 7.52 (*dd*, H, Py), 3.74 (*t*, 2H, NCH_2_), 2.64 (*t*, 2H, CH_2_S), 2.56 (*d*, H, SCH), 1.82–1.04 [*m*, 10H, (CH_2_)_5_]; ^13^C NMR (75.4 MHz, CDCl_3_): δ 166.84, 166.47, 155.60, 144.65, 139.31, 125.76, 116.76, 42.95, 37.89, 33.36, 27.71, 25.91, 25.68

## Refinement   

Crystal data, data collection and structure refinement details are summarized in Table 3 ▸. All H atoms were positioned geometrically and refined using a riding model, with C—H = 0.95 Å and *U*_iso_(H) = 1.2*U*_eq_(C) for aromatic C—H groups, C—H = 0.99 Å and *U*_iso_(H) = 1.2*U*_eq_(C) for CH_2_ groups, and C—H = 1.00 Å and *U*_iso_(H) = 1.2*U*_eq_(C) for C*sp* ^3^—H groups.

## Supplementary Material

Crystal structure: contains datablock(s) I, New_Global_Publ_Block. DOI: 10.1107/S2056989017012142/hb7685sup1.cif

Structure factors: contains datablock(s) I. DOI: 10.1107/S2056989017012142/hb7685Isup2.hkl

CCDC reference: 1570205

Additional supporting information:  crystallographic information; 3D view; checkCIF report

## Figures and Tables

**Table 1:** Hydrogen-bond geometry (Å, °)

*D*—H⋯*A*	*D*—H	H⋯*A*	*D*⋯*A*	*D*—H⋯*A*
C2—H2⋯O1^i^	0.95	2.50	3.119 (3)	123
C3—H3⋯O1^i^	0.95	2.55	3.129 (3)	119

Symmetry code: (i) 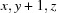.

**Table 2:** Experimental and calculated bond lengths (Å)

Bond	X-ray	B3LYP (6–311++G(d,p))	Difference
S1—C9	1.813 (3)	1.830	−0.017
S1—C10	1.827 (3)	1.853	−0.026
O1—C6	1.212 (3)	1.205	0.007
O2—C7	1.209 (3)	1.210	0.001
N1—C5	1.325 (3)	1.324	0.001
N1—C1	1.342 (4)	1.342	0.000
N2—C6	1.394 (3)	1.407	−0.013
N2—C7	1.395 (4)	1.399	−0.004
N2—C8	1.460 (3)	1.456	0.004
C1—C2	1.382 (4)	1.400	−0.018
C2—C3	1.381 (4)	1.396	−0.015
C3—C4	1.380 (4)	1.385	−0.005
C4—C5	1.376 (4)	1.392	−0.016
C4—C7	1.490 (4)	1.492	−0.002
C5—C6	1.497 (4)	1.508	−0.011
C8—C9	1.522 (4)	1.536	−0.014
C10—C11	1.516 (4)	1.534	−0.018
C10—C15	1.530 (4)	1.536	−0.006
C11—C12	1.523 (4)	1.539	−0.016
C12—C13	1.523 (4)	1.534	−0.011
C13—C14	1.514 (4)	1.535	−0.021
C14—C15	1.524 (5)	1.537	−0.013

**Table 3:** Experimental details

Crystal data	
Chemical formula	C_15_H_18_N_2_O_2_S
*M*_r_	290.37
Crystal system, space group	Orthorhombic, *P*2_1_2_1_2_1_
Temperature (K)	173
*a*, *b*, *c* (Å)	5.5322 (2), 7.8707 (3), 32.9092 (14)
*V* (Å^3^)	1432.94 (10)
*Z*	4
Radiation type	Mo *K*α
μ (mm^−1^)	0.23
Crystal size (mm)	0.28 × 0.10 × 0.09

Data collection	
Diffractometer	Bruker APEXII CCD
Absorption correction	Multi-scan (*SADABS*; Bruker, 2014 ▸)
*T*_min_, *T*_max_	0.690, 0.746
No. of measured, independent and observed [*I* > 2σ(*I*)] reflections	11035, 2536, 2302
*R*_int_	0.046
(sin θ/λ)_max_ (Å^−1^)	0.595

Refinement	
*R*[*F* ^2^ > 2σ(*F* ^2^)], *wR*(*F* ^2^), *S*	0.034, 0.072, 1.04
No. of reflections	2536
No. of parameters	181
H-atom treatment	H-atom parameters constrained
Δρ_max_, Δρ_min_ (e Å^−3^)	0.20, −0.19
Absolute structure	Flack *x* determined using 839 quotients [(*I* ^+^) − (*I* ^−^)]/[(*I* ^+^) + (*I* ^−^)] (Parsons *et al.*, 2013 ▸)
Absolute structure parameter	0.05 (5)

Computer programs: *APEX2* (Bruker, 2014 ▸), *SAINT* (Bruker, 2014 ▸), *SHELXS97* (Sheldrick, 2008 ▸), *SHELXL2014* (Sheldrick, 2015 ▸), *DIAMOND* (Brandenburg, 2010 ▸), *SHELXTL* (Sheldrick, 2008 ▸) and *publCIF* (Westrip, 2010 ▸).

## supplementary crystallographic information

### Crystal data

**Table:**

C_15_H_18_N_2_O_2_S	*D*_x_ = 1.346 Mg m^−^^3^
*M**_r_* = 290.37	Mo *K*α radiation, λ = 0.71073 Å
Orthorhombic, *P*2_1_2_1_2_1_	Cell parameters from 2024 reflections
*a* = 5.5322 (2) Å	θ = 2.5–27.2°
*b* = 7.8707 (3) Å	µ = 0.23 mm^−^^1^
*c* = 32.9092 (14) Å	*T* = 173 K
*V* = 1432.94 (10) Å^3^	Plate, colourless
*Z* = 4	0.28 × 0.10 × 0.09 mm
*F*(000) = 616	

### Data collection

**Table:**

Bruker APEXII CCD diffractometer	2302 reflections with *I* > 2σ(*I*)
φ and ω scans	*R*_int_ = 0.046
Absorption correction: multi-scan (SADABS; Bruker, 2014)	θ_max_ = 25.0°, θ_min_ = 1.2°
*T*_min_ = 0.690, *T*_max_ = 0.746	*h* = −6→6
11035 measured reflections	*k* = −8→9
2536 independent reflections	*l* = −39→39

### Refinement

**Table:**

Refinement on *F*^2^	Hydrogen site location: inferred from neighbouring sites
Least-squares matrix: full	H-atom parameters constrained
*R*[*F*^2^ > 2σ(*F*^2^)] = 0.034	*w* = 1/[σ^2^(*F*_o_^2^) + (0.0215*P*)^2^ + 0.3497*P*] where *P* = (*F*_o_^2^ + 2*F*_c_^2^)/3
*wR*(*F*^2^) = 0.072	(Δ/σ)_max_ < 0.001
*S* = 1.04	Δρ_max_ = 0.20 e Å^−^^3^
2536 reflections	Δρ_min_ = −0.19 e Å^−^^3^
181 parameters	Absolute structure: Flack *x* determined using 839 quotients [(I+)-(I-)]/[(I+)+(I-)] (Parsons *et al.*, 2013)
0 restraints	Absolute structure parameter: 0.05 (5)

### Special details

**Table:**

Geometry. All esds (except the esd in the dihedral angle between two l.s. planes) are estimated using the full covariance matrix. The cell esds are taken into account individually in the estimation of esds in distances, angles and torsion angles; correlations between esds in cell parameters are only used when they are defined by crystal symmetry. An approximate (isotropic) treatment of cell esds is used for estimating esds involving l.s. planes.

### Fractional atomic coordinates and isotropic or equivalent isotropic displacement parameters (Å^2^)

**Table:**

	*x*	*y*	*z*	*U*_iso_*/*U*_eq_	
S1	0.53243 (13)	0.93377 (11)	0.09181 (3)	0.0414 (2)	
O1	0.5825 (3)	0.9636 (2)	0.20137 (6)	0.0362 (5)	
O2	0.1331 (3)	1.3885 (3)	0.14389 (6)	0.0360 (5)	
N1	0.8186 (4)	1.2919 (3)	0.22791 (7)	0.0292 (6)	
N2	0.3211 (4)	1.1461 (3)	0.16766 (7)	0.0268 (5)	
C1	0.8794 (5)	1.4552 (4)	0.23364 (8)	0.0306 (7)	
H1	1.0179	1.4779	0.2498	0.037*	
C2	0.7561 (5)	1.5929 (4)	0.21783 (8)	0.0320 (7)	
H2	0.8111	1.7050	0.2233	0.038*	
C3	0.5532 (5)	1.5678 (3)	0.19413 (8)	0.0301 (6)	
H3	0.4639	1.6597	0.1829	0.036*	
C4	0.4884 (5)	1.4006 (3)	0.18778 (7)	0.0235 (6)	
C5	0.6241 (5)	1.2727 (3)	0.20487 (8)	0.0234 (6)	
C6	0.5174 (5)	1.1058 (3)	0.19241 (8)	0.0262 (6)	
C7	0.2900 (5)	1.3212 (4)	0.16376 (8)	0.0281 (7)	
C8	0.1681 (5)	1.0227 (4)	0.14678 (9)	0.0335 (7)	
H8A	−0.0035	1.0559	0.1499	0.040*	
H8B	0.1895	0.9095	0.1594	0.040*	
C9	0.2300 (5)	1.0119 (4)	0.10179 (9)	0.0369 (8)	
H9A	0.2130	1.1263	0.0896	0.044*	
H9B	0.1120	0.9361	0.0883	0.044*	
C10	0.4745 (5)	0.7095 (3)	0.08142 (8)	0.0278 (6)	
H10	0.3597	0.6645	0.1023	0.033*	
C11	0.3645 (5)	0.6857 (4)	0.03962 (8)	0.0318 (7)	
H11A	0.4706	0.7395	0.0191	0.038*	
H11B	0.2057	0.7435	0.0387	0.038*	
C12	0.3313 (6)	0.4988 (4)	0.02908 (10)	0.0448 (9)	
H12A	0.2117	0.4476	0.0478	0.054*	
H12B	0.2676	0.4887	0.0011	0.054*	
C13	0.5693 (6)	0.4026 (4)	0.03229 (10)	0.0463 (8)	
H13A	0.5408	0.2803	0.0271	0.056*	
H13B	0.6831	0.4450	0.0114	0.056*	
C14	0.6795 (6)	0.4253 (4)	0.07406 (10)	0.0438 (8)	
H14A	0.8382	0.3672	0.0750	0.053*	
H14B	0.5734	0.3716	0.0946	0.053*	
C15	0.7133 (5)	0.6123 (4)	0.08458 (9)	0.0388 (8)	
H15A	0.8329	0.6633	0.0658	0.047*	
H15B	0.7773	0.6223	0.1126	0.047*	

### Atomic displacement parameters (Å^2^)

**Table:**

	*U*^11^	*U*^22^	*U*^33^	*U*^12^	*U*^13^	*U*^23^
S1	0.0268 (4)	0.0445 (5)	0.0529 (5)	−0.0066 (4)	0.0022 (3)	−0.0203 (4)
O1	0.0412 (12)	0.0199 (11)	0.0474 (12)	0.0036 (10)	−0.0046 (10)	0.0019 (10)
O2	0.0354 (11)	0.0345 (12)	0.0380 (11)	0.0093 (10)	−0.0109 (9)	−0.0026 (10)
N1	0.0318 (13)	0.0227 (14)	0.0332 (13)	0.0016 (11)	−0.0039 (11)	−0.0014 (11)
N2	0.0258 (12)	0.0212 (13)	0.0333 (13)	−0.0014 (11)	−0.0006 (11)	−0.0047 (10)
C1	0.0334 (15)	0.0274 (17)	0.0310 (16)	0.0000 (14)	−0.0036 (12)	−0.0047 (13)
C2	0.0432 (17)	0.0200 (17)	0.0329 (16)	−0.0009 (14)	−0.0028 (13)	−0.0022 (13)
C3	0.0408 (15)	0.0212 (15)	0.0282 (14)	0.0072 (14)	−0.0074 (13)	−0.0001 (12)
C4	0.0302 (14)	0.0186 (14)	0.0217 (13)	0.0009 (13)	−0.0013 (11)	−0.0016 (11)
C5	0.0263 (13)	0.0186 (15)	0.0252 (14)	0.0002 (12)	0.0010 (12)	−0.0010 (12)
C6	0.0275 (14)	0.0215 (15)	0.0297 (14)	0.0013 (13)	0.0031 (12)	−0.0012 (12)
C7	0.0304 (15)	0.0289 (17)	0.0251 (14)	0.0027 (14)	0.0033 (12)	−0.0035 (13)
C8	0.0253 (14)	0.0272 (17)	0.0478 (18)	−0.0047 (13)	0.0013 (14)	−0.0099 (14)
C9	0.0290 (14)	0.0363 (18)	0.0455 (18)	0.0024 (13)	−0.0079 (13)	−0.0159 (14)
C10	0.0245 (14)	0.0323 (16)	0.0265 (14)	−0.0028 (13)	0.0030 (12)	−0.0022 (12)
C11	0.0361 (16)	0.0329 (18)	0.0265 (15)	0.0029 (14)	−0.0032 (13)	−0.0027 (13)
C12	0.0435 (19)	0.039 (2)	0.052 (2)	0.0007 (16)	−0.0084 (16)	−0.0145 (16)
C13	0.0491 (19)	0.0342 (19)	0.056 (2)	0.0052 (17)	0.0069 (16)	−0.0118 (16)
C14	0.0379 (17)	0.042 (2)	0.051 (2)	0.0103 (18)	0.0047 (15)	0.0076 (17)
C15	0.0307 (15)	0.049 (2)	0.0372 (17)	0.0036 (15)	−0.0018 (13)	−0.0023 (16)

### Geometric parameters (Å, º)

**Table:**

S1—C9	1.813 (3)	C8—H8B	0.9900
S1—C10	1.827 (3)	C9—H9A	0.9900
O1—C6	1.212 (3)	C9—H9B	0.9900
O2—C7	1.209 (3)	C10—C11	1.516 (4)
N1—C5	1.325 (3)	C10—C15	1.530 (4)
N1—C1	1.342 (4)	C10—H10	1.0000
N2—C6	1.394 (3)	C11—C12	1.523 (4)
N2—C7	1.395 (4)	C11—H11A	0.9900
N2—C8	1.460 (3)	C11—H11B	0.9900
C1—C2	1.382 (4)	C12—C13	1.523 (4)
C1—H1	0.9500	C12—H12A	0.9900
C2—C3	1.381 (4)	C12—H12B	0.9900
C2—H2	0.9500	C13—C14	1.514 (4)
C3—C4	1.380 (4)	C13—H13A	0.9900
C3—H3	0.9500	C13—H13B	0.9900
C4—C5	1.376 (4)	C14—C15	1.524 (5)
C4—C7	1.490 (4)	C14—H14A	0.9900
C5—C6	1.497 (4)	C14—H14B	0.9900
C8—C9	1.522 (4)	C15—H15A	0.9900
C8—H8A	0.9900	C15—H15B	0.9900
			
C9—S1—C10	101.54 (14)	H9A—C9—H9B	107.7
C5—N1—C1	113.2 (2)	C11—C10—C15	110.3 (2)
C6—N2—C7	112.0 (2)	C11—C10—S1	111.1 (2)
C6—N2—C8	125.1 (2)	C15—C10—S1	108.6 (2)
C7—N2—C8	122.8 (2)	C11—C10—H10	109.0
N1—C1—C2	125.0 (3)	C15—C10—H10	109.0
N1—C1—H1	117.5	S1—C10—H10	109.0
C2—C1—H1	117.5	C10—C11—C12	112.0 (2)
C3—C2—C1	120.1 (3)	C10—C11—H11A	109.2
C3—C2—H2	120.0	C12—C11—H11A	109.2
C1—C2—H2	120.0	C10—C11—H11B	109.2
C4—C3—C2	115.7 (3)	C12—C11—H11B	109.2
C4—C3—H3	122.2	H11A—C11—H11B	107.9
C2—C3—H3	122.2	C13—C12—C11	111.1 (3)
C5—C4—C3	119.6 (2)	C13—C12—H12A	109.4
C5—C4—C7	108.2 (2)	C11—C12—H12A	109.4
C3—C4—C7	132.2 (2)	C13—C12—H12B	109.4
N1—C5—C4	126.4 (2)	C11—C12—H12B	109.4
N1—C5—C6	125.3 (2)	H12A—C12—H12B	108.0
C4—C5—C6	108.3 (2)	C14—C13—C12	110.6 (3)
O1—C6—N2	125.7 (2)	C14—C13—H13A	109.5
O1—C6—C5	128.7 (2)	C12—C13—H13A	109.5
N2—C6—C5	105.5 (2)	C14—C13—H13B	109.5
O2—C7—N2	124.8 (3)	C12—C13—H13B	109.5
O2—C7—C4	129.2 (3)	H13A—C13—H13B	108.1
N2—C7—C4	106.0 (2)	C13—C14—C15	111.7 (3)
N2—C8—C9	111.4 (2)	C13—C14—H14A	109.3
N2—C8—H8A	109.4	C15—C14—H14A	109.3
C9—C8—H8A	109.4	C13—C14—H14B	109.3
N2—C8—H8B	109.4	C15—C14—H14B	109.3
C9—C8—H8B	109.4	H14A—C14—H14B	107.9
H8A—C8—H8B	108.0	C14—C15—C10	111.2 (3)
C8—C9—S1	113.7 (2)	C14—C15—H15A	109.4
C8—C9—H9A	108.8	C10—C15—H15A	109.4
S1—C9—H9A	108.8	C14—C15—H15B	109.4
C8—C9—H9B	108.8	C10—C15—H15B	109.4
S1—C9—H9B	108.8	H15A—C15—H15B	108.0
			
C5—N1—C1—C2	0.3 (4)	C6—N2—C7—C4	1.1 (3)
N1—C1—C2—C3	0.1 (4)	C8—N2—C7—C4	−177.0 (2)
C1—C2—C3—C4	−0.3 (4)	C5—C4—C7—O2	−179.3 (3)
C2—C3—C4—C5	0.2 (4)	C3—C4—C7—O2	−0.8 (5)
C2—C3—C4—C7	−178.2 (3)	C5—C4—C7—N2	−0.6 (3)
C1—N1—C5—C4	−0.4 (4)	C3—C4—C7—N2	177.9 (3)
C1—N1—C5—C6	178.4 (3)	C6—N2—C8—C9	−103.0 (3)
C3—C4—C5—N1	0.2 (4)	C7—N2—C8—C9	74.9 (3)
C7—C4—C5—N1	179.0 (2)	N2—C8—C9—S1	64.2 (3)
C3—C4—C5—C6	−178.8 (2)	C10—S1—C9—C8	97.4 (2)
C7—C4—C5—C6	0.0 (3)	C9—S1—C10—C11	75.0 (2)
C7—N2—C6—O1	178.8 (3)	C9—S1—C10—C15	−163.5 (2)
C8—N2—C6—O1	−3.1 (4)	C15—C10—C11—C12	55.5 (3)
C7—N2—C6—C5	−1.1 (3)	S1—C10—C11—C12	175.9 (2)
C8—N2—C6—C5	176.9 (2)	C10—C11—C12—C13	−56.0 (4)
N1—C5—C6—O1	1.7 (4)	C11—C12—C13—C14	55.3 (4)
C4—C5—C6—O1	−179.3 (3)	C12—C13—C14—C15	−55.8 (4)
N1—C5—C6—N2	−178.4 (2)	C13—C14—C15—C10	56.0 (3)
C4—C5—C6—N2	0.6 (3)	C11—C10—C15—C14	−55.2 (3)
C6—N2—C7—O2	179.9 (3)	S1—C10—C15—C14	−177.1 (2)
C8—N2—C7—O2	1.8 (4)		

### Hydrogen-bond geometry (Å, º)

**Table:**

*D*—H···*A*	*D*—H	H···*A*	*D*···*A*	*D*—H···*A*
C2—H2···O1^i^	0.95	2.50	3.119 (3)	123
C3—H3···O1^i^	0.95	2.55	3.129 (3)	119

Symmetry code: (i) *x*, *y*+1, *z*.
